# Calorie restriction does not influence oocyte quality in oocytes from POLG mitochondrial mutator mice

**DOI:** 10.1371/journal.pone.0204373

**Published:** 2018-09-21

**Authors:** Christine Faraci, Joyce Jin, Dori C. Woods

**Affiliations:** Laboratory for Aging and Infertility Research, Department of Biology, Northeastern University, Boston, MA, United States of America; University of Parma, ITALY

## Abstract

It has recently been demonstrated that moderate adult onset caloric restriction (*e*.*g*. calorie restriction; CR) has a positive impact on female fertility in aged mice, due in large to preventing the age-associated decline in the quality of oocytes available for fertilization. The impact of CR on oocyte quality has been attributed, at least in part, to mitochondrial functions. In mitochondrial DNA (mtDNA) mutator mice (*Polg*^D257A/D257A^), which harbor a mutation in the proofreading mtDNA polymerase-gamma (POLG), mitochondrial mutations rapidly accumulate, resulting in a premature aging phenotype and female infertility. As CR has been shown to extend both lifespan and ‘healthspan’ as well as improve oocyte quality in aged mice, we investigated whether adult onset CR could improve oocyte quality in the POLG mouse. Female *Polg*^D257A/D257A^ mice exhibited infertility based on an inability to produce litters through natural mating. Analysis of oocytes from 8–9-month-old *Polg*^D257A/D257A^ mice on CR following hormone stimulation revealed no improvement in the number of oocytes ovulated. Furthermore, CR did not result in a greater percentage of metaphase II oocytes, with the majority of the oocytes prematurely arrested at the germinal vesicle stage. Finally, CR did not improve the abnormal mitochondrial distribution or pronounced defects in meiotic spindle assembly and chromosomal distribution observed in the ad libitum fed *Polg*^D257A/D257A^. Taken together, these data suggest that although CR benefits oocyte quality and fertility outcomes in naturally aged female mice, it does not sufficiently improve oocyte quality in *Polg*^D257A/D257A^.

## Introduction

The *Polg*^D257A/D257A^, or POLG mitochondrial DNA (mtDNA) mutator mouse harbors a D257A mutation in the exonuclease domain of the gene encoding the sole mtDNA ‘proofreading’ polymerase, polymerase γ (Polg) [1–3]. As a result, the homozygous mutant protein is rendered devoid of proofreading function, leading to an accelerated accumulation of somatic mtDNA mutations and a progeroid phenotype [2–4]. Accordingly, there have been intense investigative efforts utilizing *Polg*^D257A/D257A^, aimed at elucidating the role of mtDNA mutations as a potential driving force in the aging process and related aging phenotypes [4–9]. Among the characteristics aligned with those that accompany naturally aging mice, *Polg*^D257A/D257A^ exhibit alopecia, anemia, reduced subcutaneous fat, kyphosis, and osteoporosis by approximately 6 months of age, with a truncated life-span of approximately 12 months-of-age [2, 3]. Additionally, both sexes of *Polg*^D257A/D257A^ mice have fertility defects, with male mice exhibiting small testicular size and reduced sperm production by 12 weeks of age, followed by testicular atrophy and complete loss of sperm by 40 weeks-of-age [3]. The exact mechanisms driving the specific fertility defect in the female *Polg*^D257A/D257A^ have not been elucidated.

Intriguingly, it has been reported that endurance exercise ameliorates a spectrum of characteristics associated with the progeroid phenotype in *Polg*^D257A/D257A^ and extends lifespan in these animals [10–12]. However, our recent work demonstrates that unlike the impact of exercise in the soma, endurance exercise confers little benefit to oocytes, and does not extend or enhance female fertility in *Polg*^D257A/D257^[13], which is consistent with other recent work demonstrating only a modest impact of exercise on the mitochondria of oocytes in mice fed a high fat diet [14]. However, unlike exercise, it has been demonstrated that adult onset calorie restriction prevents the spindle defects, chromosomal misalignments, and abnormal mitochondrial distribution that occur in female mice with age [15]. Herein we investigated whether the benefit of CR that has been observed in aged WT could similarly prevent deficits in oocyte quality attributed to the elevated levels of mtDNA mutations and accelerated aging phenotype in female *Polg*^D257A/D257A^.

## Materials and methods

### Animals and calorie restriction

Heterozygous male mice (Polg ^D257A/+^) obtained from the Jackson Laboratory were mated with WT females to ‘reset’ the mutational background. Polg ^D257A/+^ breeding pairs were then used to generate the homozygous knock-in mtDNA mutator mice (Polg^D257A/257A^). Unless otherwise stated, Polg^D257A/257A^ were used for each experiment. All experiments described herein were reviewed and approved by the Institutional Animal Care and Use Committee (IACUC) of Northeastern University.

For CR, we utilized a protocol developed by the National Institute on Aging for adult onset CR Calorie [16], following the feeding regime essentially as described in [15]. Female Polg^D257A/257A^ mice were housed individually in pathogen-free facilities with free access to food and water. At 3 months of age, Polg^D257A/D257A^ were randomly assigned to ad libitum (AL) or CR groups, with free access to water, for the duration of the study. CR was initiated in a stepwise manner over a period of 2 weeks to obtain a final 40% restriction of fortified rodent diet, containing micronutrients comparable to a standard rodent diet (National Institute on Aging). Body weight and condition were analyzed weekly throughout the study.

### Oocyte retrieval

Intraperitoneal injection of pregnant mare serum gonadotropin (PMSG, 10IU; Sigma-Aldrich, St. Louis, MO, USA) followed by human chorionic gonadotropin (hCG, 10IU; Sigma-Aldrich; 46–48 h post-PMSG) was used to induce ovulation in female Polg^D257A/257A^ mice between 5 and 10 months-of-age, as described previously [13]. 15–16 h post hCG injection, mice were euthanized by CO_2_ asphyxiation followed by secondary cervical dislocation, and the ovaries and attached oviducts were collected for oocyte retrieval. Cumulus oocyte complexes (COCs) were released from the oviducts following puncturing the oviducts with a fine needle. Collected COCs were denuded of cumulus cells by incubating for 2 min in 80 IU mL^−1^ of hyaluronidase (Sigma-Aldrich) at 37°C, followed by three washes with human tubal fluid (HTF; Irvine Scientific, Santa Ana, CA, USA) supplemented with 0.4% BSA (fraction V, fatty acid free; Sigma-Aldrich) at 37°C. Oocytes were counted and classified using a Zeiss Stereo Microscope (Zeiss, USA) as metaphase II (MII; extrusion of the first polar body into the perivitelline space), maturation arrested (germinal vesicle stage, or germinal vesicle breakdown without polar body extrusion), or atretic (membrane blebbing or condensed cytoplasm). Following analysis, oocytes were fixed in 2% paraformaldehyde (PFA) for 30 min at 37°C for endpoint analyses.

### Mitochondrial distribution analysis

Following fixation, oocytes were incubated in permeabilization buffer [1% bovine serum albumen (BSA), 5% normal goat serum (NGS), 0.1% Triton-X, 0.05% Tween-20 in PBS) for 30 minutes, as described previously [13]. Mitochondria were then labeled with 500nM Mitotracker Red CMXros for 1 h at room temperature. Once labeled, oocytes were washed in phosphate-buffered saline (PBS; Sigma-Aldrich) and then mounted and imaged at 63x magnification on a laser scanning confocal microscope (Zeiss). Normal mitochondrial distribution was defined by a uniform and punctate cytoplasmic distribution, whereas oocytes containing mitochondria having diffuse or condensed patterning were classified as abnormal.

### Immunofluorescence

Fixed oocytes were incubated in permeabilization buffer for 30 minutes, followed by a brief wash in PBS and incubation in blocking buffer (2% BSA, 2% NGS in PBS) for 1 h. A 1:100 dilution of mouse anti α-tubulin antibody (Sigma-Aldrich) was added to the sample, and incubated for 1 h, followed by 3 washes for 5 min each in PBS. The samples were then incubated with goat anti-mouse secondary antibody, conjugated to Alexa-488 (1:500; Life Technologies, Carlsbad, CA). The immunolabeled oocytes were then washed 3 times in PBS for 5 min, with DAPI (1:100) added during the final wash step for visualization of DNA. Oocytes were then mounted and analyzed by confocal microscopy for spindle morphology and chromosome alignment. Meiotic spindles with a proper barrel shape and attachment to chromosomes were scored as normal, while missing spindles, spindles that failed to attach to chromosomes, and spindles that did not have clear centrioles were marked as abnormal. Chromosome segregation was scored as normal if all chromosomes were lined in a linear fashion, while scattering of chromosomes was scored as abnormal, as described previously [13].

### Statistical analysis

Statistical comparisons were made between groups from experimental replicates, which were combined and presented as the mean ± s.e.m. or mean ± standard deviation (s.d.) of binomial distribution. Statistical significance was determined using Student’s *t*-test, with *P* values < 0.05 considered significant. Statistical comparisons between more than three groups were performed using ANOVA, followed by Tukey’s *t*-test (*P* < 0.05 considered significant).

## Results

**Fig 1:**
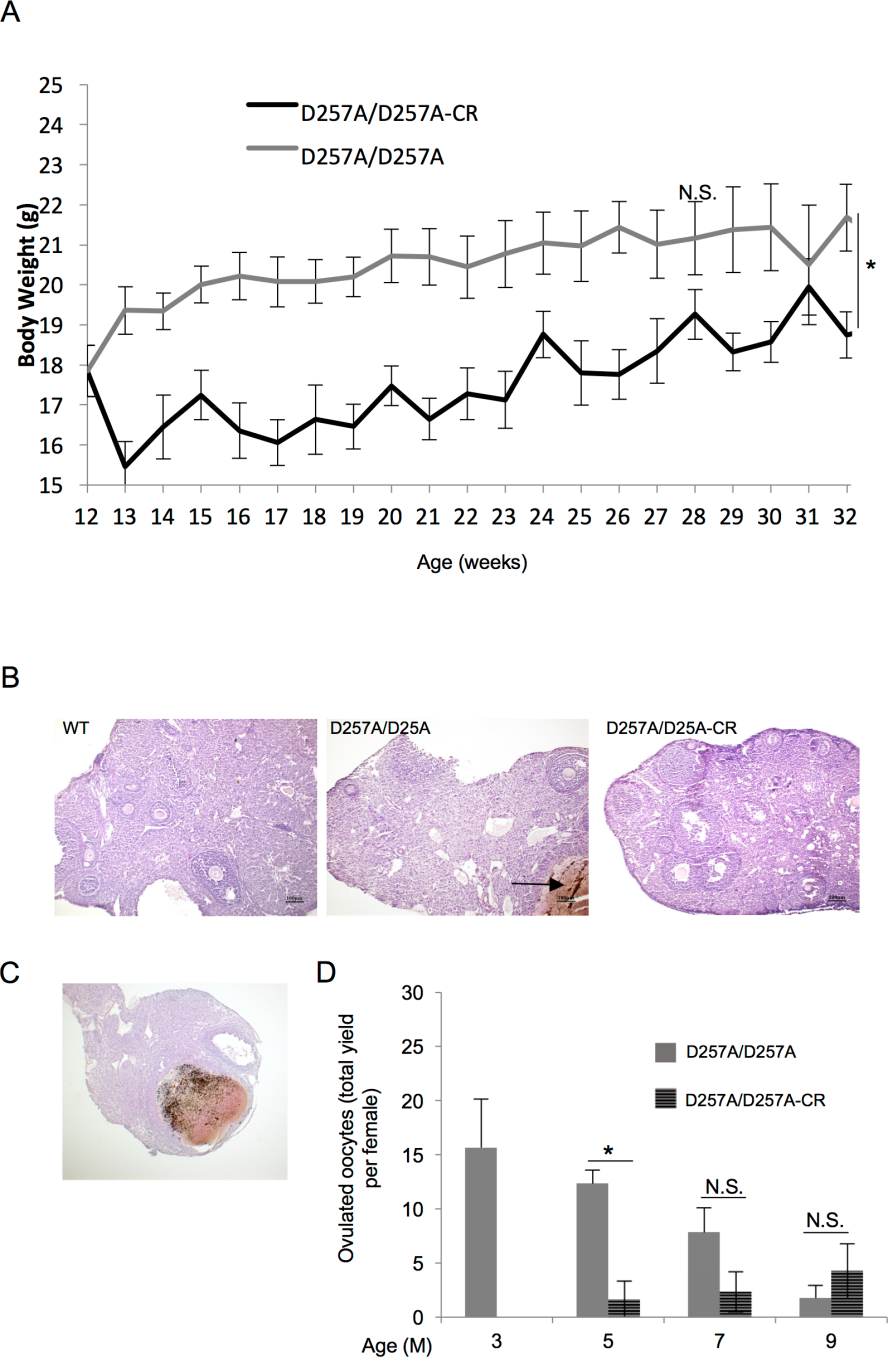
Body weight and ovarian characteristics following CR in POLG^D257A/257A^ female mice. **A.** Body weight was decreased following the onset of CR. AL-fed POLG^D257A/257A^ mice (D57A/D57A) denoted by grey line; POLG on a CR diet (D57A/D57A-CR) denoted by black line (error bars shown at selected age intervals for clarity, n = 3–10 mice per group, * indicates *P* < 0.05, N.S., not significant). **B.** Histological sections of representative ovaries from 7-month old WT, AL-fed POLG^D257A/257A^, and POLG^D257A/257A^ fed a CR diet demonstrates normal ovarian follicle distribution. Of note, ovarian cysts were present in approximately 50% ovaries collected from POLG^D257A/257A^ mice, regardless of dietary status (ovarian cyst denoted by black arrow; ovaries from 4 out of 8 POLG^D257A/257A^ and 5 out of 8 POLG^D257A/257A^-CR harbored cysts). **C.** Representative histological ovarian section of a POLG^D257A/257A^ mouse containing a large ovarian cyst. **D.** CR reduces the number of ovulated oocytes at 5 and 7 months of age in POLG^D257A/257A^ mice (3 months n = 3; 5-, 7-, 9-month n = 6).

As anticipated, throughout the duration of the study, Polg^D257A/257A^ maintained on a CR diet sustained a significantly reduced body weight as compared to AL Polg^D257A/257A^ females (Fig 1A). Gross histological examination of ovarian tissue collected at 7 months of age, prior to the near-complete ovarian failure that occurs by 9-months of age, revealed follicles from multiple stages of development in both AL and CR Polg^D257A/257A^ (Fig 1B). Although ovarian morphology was essentially normal with regard to follicle formation, there was a notable increase in ovarian cysts found in the ovaries of Polg^D257A/257A^, regardless of CR or AL status, as compared to WT counterparts (Fig 1B and 1C), ovaries from 4 out of 8 POLG^D257A/257A^ and 5 out of 8 POLG^D257A/257A^-CR harboring cysts. We also found that, as anticipated, mice continued on CR exhibited a reduction in the number of oocytes ovulated per female as compared to both Polg^D257A/257A^ AL fed mice, as well as WT controls, with a slight, although not statistically significant, increase in the number of oocytes ovulated at 9-months of age (Fig 1D). This reduction in the number of the ovulated oocytes is consistent with the documented suppression of ovulation by CR [17, 18].

**Fig 2:**
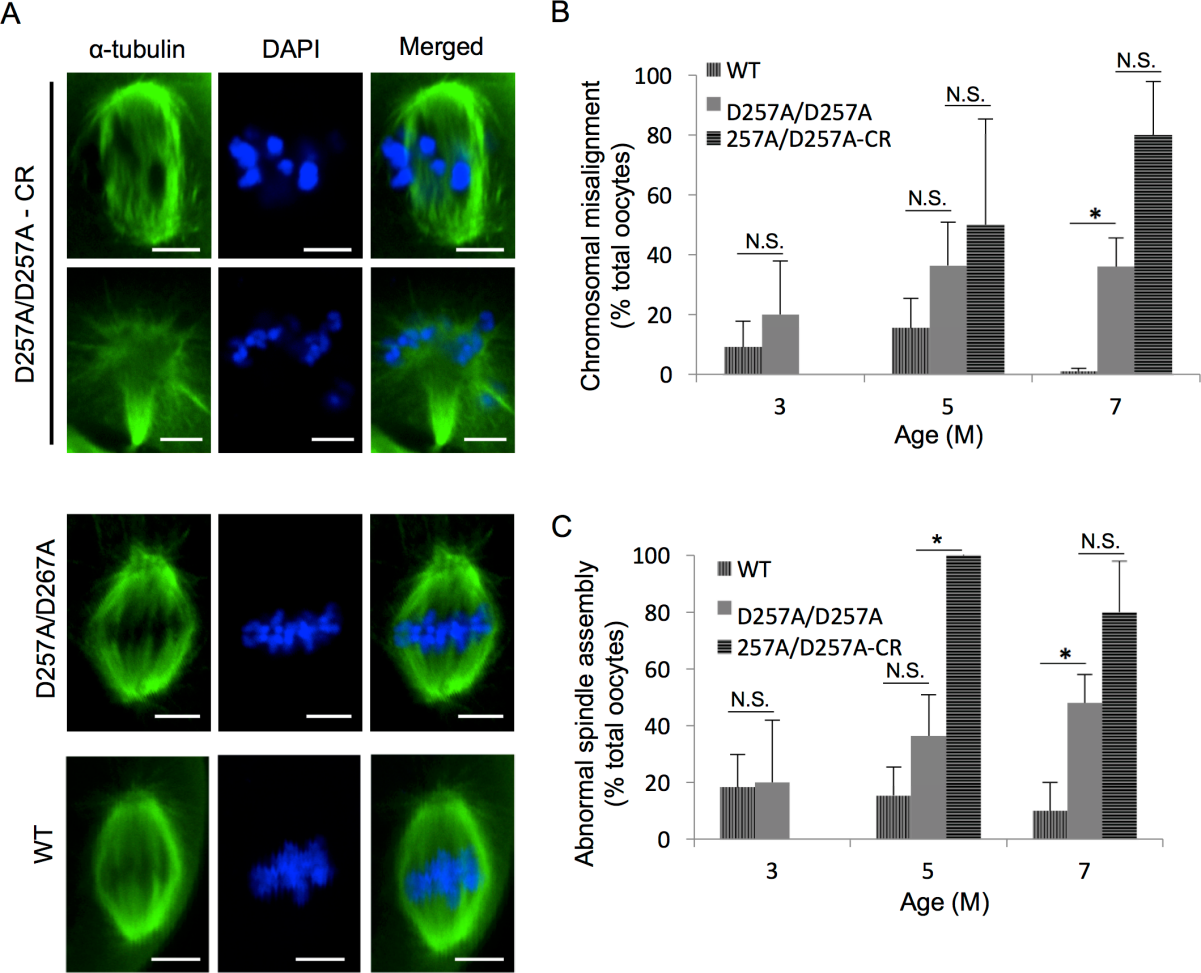
CR does not improve chromosome alignment or meiotic spindle assembly in oocytes from POLG^D257A/257A^ mice. **A.** Representative laser scanning confocal micrographs of meiotic spindles in MII oocytes from WT, 7-month-old POLG^D257A/257A^, and POLG^D257A/257A^-CR mice; α-tubulin labeling of spindle (green) and DNA labeled with DAPI (blue). Scale bars = 5 μm. **B, C.** Percentage of chromosome misalignment and abnormal spindle assembly in oocytes from WT, POLG^D257A/257A^, and POLG^D257A/257A^-CR mice collected at 3–9 months of age (mean shown, error calculated as SD of a binomial distribution; only MII analyzed, oocytes were from 3–6 mice per group; N.S., not significant; *P* > 0.05; * *P* < 0.05).

To determine if CR improves the quality of oocytes from Polg^D257A/257A^ mice post-ovulation as has been previously reported in WT female mice [15], we analyzed meiotic spindle assembly and chromosomal alignment in ovulated oocytes, with arrested and atretic oocytes excluded from analysis. We found that while both abnormal spindle assembly and chromosomal misalignment increased with age in Polg^D257A/257A^, CR did not abrogate the increase in spindle defects nor chromosomal abnormalities (Fig 2A and 2B). Interestingly, we found that CR enhanced the instance of spindle defects observed in Polg^D257A/257A^ oocytes as early as 5 months of age, and the rate of spindle abnormalities was maintained until 9-months of age (Fig 2C).

**Fig 3:**
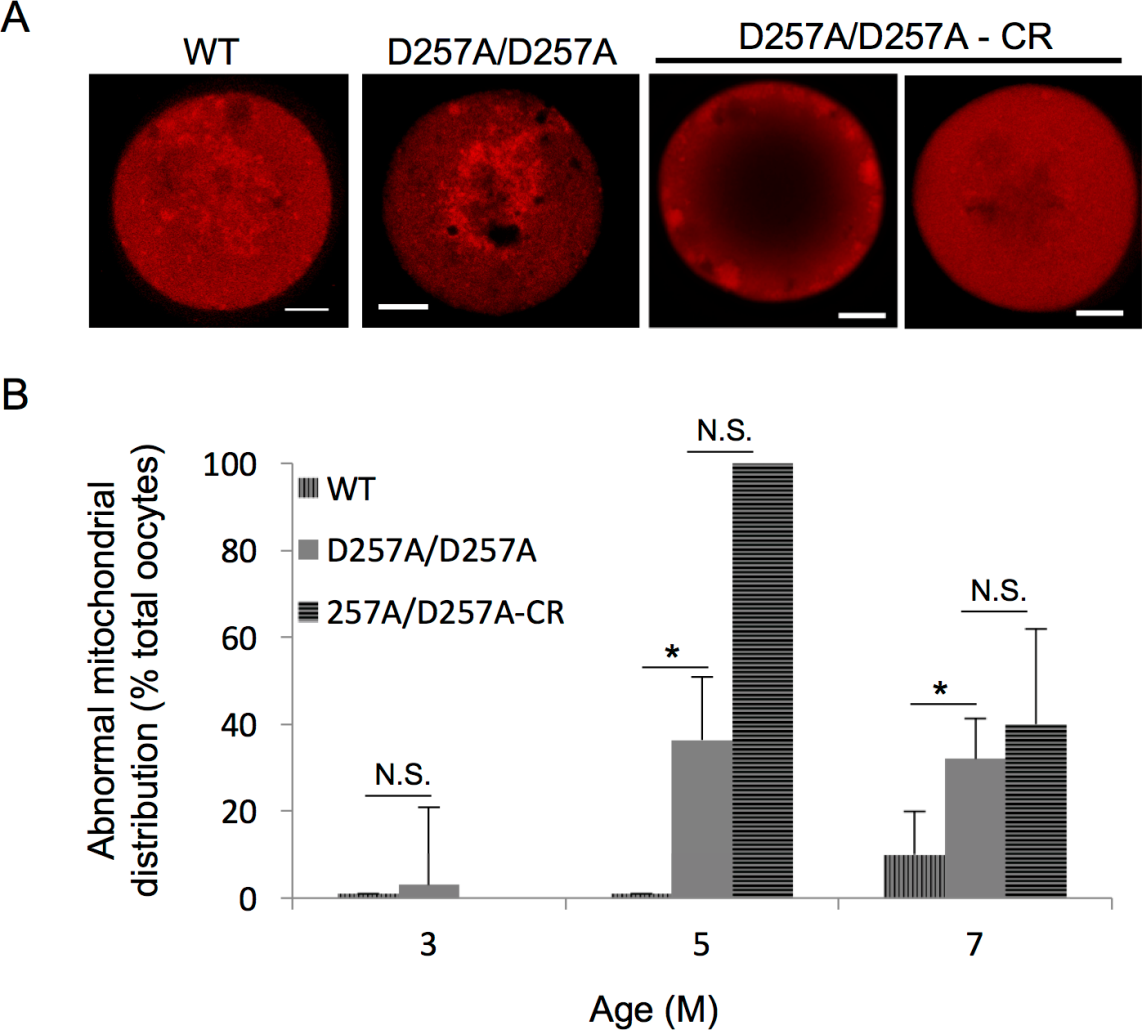
CR does not improve mitochondrial distribution in oocytes from POLG^D257A/257A^. **A.** Representative images of MII eggs labeled with MitoTracker (shown in red) from 7-month-old WT, POLG^D257A/257A^, and POLG^D257A/257A^-CR mice. Abnormal mitochondrial clustering was prevalent in POLG^D257A/257A^, whereas oocytes collected from POLG^D257A/257A^-CR mice demonstrated diffuse mitochondrial labeling. **B.** Percentage of abnormal mitochondrial distribution in MII oocytes collected from WT, POLG^D257A/257A^, and POLG^D257A/257A^-CR mice (mean shown, error calculated as SD of a binomial distribution; only MII analyzed, oocytes were from 3–6 mice per group; N.S., not significant; *P* > 0.05).

Finally, it is well understood that mitochondrial distribution within oocytes becomes perturbed with age in WT mice, and it is thought to contribute to the age-associated decline in infertility [14, 15]. To determine whether mitochondrial perturbation in oocytes was curbed by CR restriction in Polg^D257A/257A^ mice, mitochondrial distribution patterns were evaluated (Fig 3A and 3B). LSC microscopy revealed clumped mitochondria in Polg^D257A/257A^, consistent with what has previously been reported in oocytes obtained from aged mice (Selesniemi *et al*. 2011), however the oocytes obtained from Polg^D257A/257A^ mice on a CR diet had diffuse mitochondrial staining, with little to no punctate foci, or irregular aggregation on the periphery, with no perinuclear localization. Although CR did not improve the abnormal mitochondrial distribution observed the oocytes isolated from Polg^D257A/257A^ female mice, irregularities in mitochondrial distribution were morphologically distinct from those normally associated with an aging phenotype. The lack of perinuclear organization and prevalence of diffuse mitochondrial patterning as a result of CR is similar to that observed in immature oocytes, or those that fail to properly progress through meiosis (reviewed in [19].

## Discussion

Our study demonstrates that, unlike CR in WT mice, CR does not extend the fertile lifespan in POLG mice. The benefits from CR are well-documented, with CR known to extend lifespan in rodent models, as well as reduce the instance of tumor formation, maintain T-lymphocyte proliferation, and increase metabolic efficiency [20]. It is thought that the positive impact of CR is mechanistically due, in part, to improvement in mitochondrial function, including enhanced mitochondrial biogenesis, oxygen consumption and ATP synthesis, and increased expression of sirtuin 1 (SIRT1) [21]. Importantly, to date, CR has been the only intervention demonstrated to prevent or delay the onset of poor oocyte quality in rodent models of ovarian aging. Remarkably, adult onset CR leads to significant improvement in meiotic spindle assembly, chromosomal alignment, and mitochondrial distribution as compared to AL-fed counterparts [15]. However, whether or not the benefit(s) of CR extends to the POLG mitochondrial mutator mouse have only recently been explored [22]. Unlike the endurance exercise, which has been demonstrated to have a remarkable benefit on preventing the progeroid aging phenotype of the POLG mutator mouse, thought to be mediated through a p53-dependent mechanism [10–12], recent evidence demonstrates that CR does not result in abrogation of the progeroid phenotype in the POLG mouse [22]. To the contrary, data demonstrate that while CR does reduce body weight in POLG mice (similar to results reported herein), it does not improve reduction in muscle mass as compared to AL-fed counterparts, does not alter the rate of mitochondrial deletions in cardiac muscle, improve cardiac output, or extend lifespan [22]. This is attributed, at least in part, to specific differences in both metabolism and the mechanisms that result in accumulation of mtDNA defects with age between WT models and the POLG mutator mouse model. For example, during the normal aging process, oxidative stress results in accumulation of mtDNA damage or deletions [23]. CR prevents oxidative stress, reduces reactive oxygen species (ROS), and decreases mtDNA damage [24–26]. By comparison, the increased mutational burden in POLG mutator mice is due to a defect in the mtDNA polymerase proofreading function, which results in accelerated accumulation of mtDNA mutations attributed to replication errors. Accordingly, reduction of oxidative stress may not significantly abrogate mtDNA mutations in the POLG model [22].

However, the mechanisms that govern mitochondrial function in the female germline differ from those of somatic tissues. For example, unlike somatic cells and tissues, PGC1-α does not appear to be critical for maintenance of mitochondrial integrity and biogenesis on oocytes. In addition to an extended fertile period, female mice deficient in PGC1-α exhibit markedly improved oocyte quality, including reduction in abnormal mitochondrial aggregation, as well as a decrease in defects in meiotic spindle assembly and chromosomal misalignment as compared to age-matched WT counterparts [15]. Moreover, mitochondrial biogenesis ceases in oocytes prior to fertilization, with resumption of biogenesis occurring following the blastocyst stage of embryonic development. In somatic cells, mitochondrial autophagy is thought to be a critical regulatory element for maintenance of mitochondrial integrity [27], however in oocytes mitochondrial autophagy is thought to be absent [14]. Finally, our recent work demonstrates that unlike somatic tissues, in which the POLG premature aging phenotype is dramatically improved following exercise, exercise has little to no impact on the female germline or accelerated progression of infertility in these mice [13].

The present study demonstrates that, in agreement with recent data from somatic tissues, oocyte quality in the POLG mutator mouse is not improved following CR. Although the mechanisms that govern mitochondrial function deviate dramatically from those in somatic cells, these data provide additional evidence supporting that the physiological benefits of CR that occur in normal animals are not applicable to the POLG mouse [22], highlighting specific differences in the mitochondrial biology between normal aging and the progeroid phenotype that occurs as a result of accelerated accumulation of mtDNA mutations, and increased mtDNA mutational burden. It has also been suggested, in addition to differences between accumulated mtDNA damage due to oxidative stress *versus* accrual of mutations during mtDNA replications, that mice exhibiting progeroid-like syndromes may have altered mitochondrial and metabolic functions that are not subject to the mechanisms of action of CR [22]. Our findings suggest that this is likely the case in oocytes as well, as CR has been demonstrated to dramatically improve oocyte quality in naturally aged mice [15]. Oxidative stress has been demonstrated to negatively impact oocyte quality and leads to poor fertility outcomes in women [28], however it is not known whether ovaries or oocytes in POLG mutator mice harbor increased ROS or are subject to increased oxidative damage, although ROS is elevated in kidney, liver, heart and skeletal muscle of POLG mutator mice as compared to age-matched, 35–42 week-old, WT controls [29]. Given the specialized mechanisms involved in the governance of mitochondrial function in the female germline, future studies aimed at elucidating the molecular and metabolic features of the POLG mutator mouse should take oocytes into special consideration. Additional work will likely further establish mitochondrial properties unique to oocytes, and provide valuable insight into the regulatory mechanisms and key features of mitochondria that ultimately govern both oocyte quality, as well as maintenance of the germline.

## Supporting information

S1 File Data set. (XLSX)
